# Attitudes towards influenza, and COVID-19 vaccines during the COVID-19 pandemic among a representative sample of the Jewish Israeli population

**DOI:** 10.1371/journal.pone.0255495

**Published:** 2022-02-11

**Authors:** Yasmin Maor, Shaked Caspi

**Affiliations:** 1 Infectious Disease Unit, Wolfson Medical Center, Holon, Israel; 2 Faculty of Medicine, Tel Aviv University, Tel Aviv, Israel; 3 School of Computer Science and School of Mathematical science, Tel Aviv University, Tel Aviv, Israel; University of Haifa, ISRAEL

## Abstract

**Background:**

Vaccine hesitancy is increasing. We assessed attitudes toward influenza and COVID-19 vaccines and the relation between hesitancy to influenza vaccine and hesitancy towards COVID-19 vaccines.

**Methods:**

A structured questionnaire administered during September 2020 to a representative sample of the Jewish Israeli population assessed attitudes and acceptance of influenza and COVID-19 vaccines. Factors for vaccine hesitancy were determined using logistic regression. Questionnaires were administered prior to the release of clinical data regarding efficacy and safety of COVID-19 vaccines and prior to vaccine rollout.

**Results:**

We approached 10,625 people, of these 2,080 responded (19%), and 2,024 completed the questionnaire (97.3%), 64.9% aged 15–64 years and 35.1% aged ≥65 years. 37% had co-morbidities. 43.5% experienced financial deterioration due to the pandemic. 65.9% received influenza vaccine ≥1 time in the past. Influenza vaccination rates were higher in the elderly (81.8%). Reasons for influenza vaccine hesitancy were opinions that the vaccine is ineffective (27.1%), and fear of side effects (29.3%). 8.2% of people aged 16–64 and 13.8% of people aged≥65 refused to be vaccinated at least once over the course of one’s lifetime. Percent of responders willing to receive a COVID-19 vaccine were higher than percent of responders willing to receive the influenza vaccine both in people aged 16–64 years (942 (72.3%) vs. 38.4%, respectively) and in people 65 years and older (84.0% vs. 76.8%, respectively). Hesitancy towards COVID-19 vaccine was associated with hesitancy towards other vaccines. Only 26.8% would participate in a COVID-19 vaccine trial.

**Conclusions:**

Willingness to receive COVID-19 vaccine was higher than willingness to receive influenza vaccine. The results point to areas of fear from influenza vaccines side effects and lack of knowledge regarding influenza vaccines effectiveness that can be addressed to increase acceptance. Hesitancy towards other vaccines was associated with hesitancy towards COVID-19 vaccination.

## Introduction

Influenza viruses cause seasonal illness worldwide and are associated with 0.1–0.5% mortality [1]. Complication and mortality increase in patients over 70 years old, in patients with co-morbidities, in infants, pregnant women and in people suffering from obesity [2, 3].

Influenza virus is remarkable for its high rate of mutation [4]. Therefore, new vaccines are produced each year to match circulating viruses. The decision of which influenza antigens to include in the vaccines is made in advance of the influenza season and is based on global surveillance of influenza viruses circulating at the end of the prior influenza season [5]. Mismatches between the vaccine strains and the circulating strains that result in reduced efficacy of the vaccine do occur.

Coronavirus disease 2019 (COVID-19) is a highly infectious pneumonia caused by severe acute respiratory syndrome coronavirus 2 virus (SARS-CoV-2). As of December 2019, it has caused a pandemic outbreak that was first discovered in Wuhan, China [6]. Up to August 30^th^, 2020, SARS-CoV-2 has infected more than 25 million people and more than 800,000 deaths have been reported. By August 30^th^, in Israel, 110,863 people were infected, representing 12,808 cases per 1 million population and 885 people have died representing 102 deaths per 1 million population [7].

In the absence of an effective and recommended established therapy, treatment of COVID-19 has mainly been empirical and experimental in addition to supportive care. Recent observational and randomized studies, involving patients with COVID-19 admitted to the hospital demonstrated mixed results regarding the efficacy of various antiviral and antimalarial drugs [8–11]. Several vaccines have recently received emergency authorizations, but in many countries’ vaccine availability is limited [12].

Vaccines are an important way to control seasonal and pandemic influenza and are thought to be crucial in controlling the COVID-19 pandemic. In recent years, there has been a decline in the willingness to receive various vaccinations, including vaccines against influenza and pneumonia [13]. This is also true for the Israeli population [14]. In response, the World Health Organization (WHO) identified vaccine hesitancy as one of the top ten global health threats in 2019.

Reasons for the declining acceptance of vaccines include the decreased efficacy in some years of the influenza vaccine, concerns about potential side-effects from vaccines, belief that a vaccine can cause the disease it was meant to prevent, thoughts that alternative practices could eliminate the need for vaccines, conspiracy theories promoted in social networks claiming that physicians benefit financially from advocating vaccine use, and that Pharma companies advertise false information regarding vaccines. Also, some religious groups, including some Orthodox Jewish courts reject vaccine use [13, 15].

Vaccine hesitancy towards influenza vaccine is common worldwide [16, 17]. Information regarding COVID-19 vaccine hesitancy is emerging both in the general population and in health care workers [18, 19].

In the winter of 2020–2021 there were no reports of influenza in Israel probably due to COVID-19 lockdowns, wearing masks, social distancing, and limited international travel (personal communications with the Israeli ministry of health). It is reasonable to assume that in the coming winter season (2021–2022) physicians will have to deal with both COVID-19 cases together with influenza, and other winter viral infections, and the rise in pneumonia cases typical to the winter months due to opening of international travel and decrease in social distancing restrictions. Therefore, it is important to understand the effect of the current COVID-19 pandemic on the willingness of people to receive vaccines against influenza, and to assess vaccine barriers for the coming COVID-19 vaccines.

The aim of the study was to assess attitudes towards influenza vaccine, and the upcoming COVID-19 vaccines and to assess whether the current COVID-19 pandemic affects the attitudes towards influenza vaccines in a representative sample of the Jewish Israeli population. We included only Israelis identifying as Jewish and excluded ethnicities that we could not adequately represent and control for selection bias [20, 21]. While not inclusive of all ethnicities in Israel, our focus on the Jewish Israeli population provides important insights about hesitancy towards vaccines in the largest population in Israel 73.9% of the Israeli population) [22]. Our hypothesis was that hesitancy towards influenza vaccines, or other vaccines, would be associated with hesitancy towards COVID-19 vaccines. We also hypothesized that as there is more information regarding the safety and efficacy of influenza vaccines compared to COVID-19 vaccines, responders would be more inclined to receive influenza vaccines compared to COVID-19 vaccines.

## Methods

### Setting

A structured questionnaire assessing people’s attitudes towards, and acceptance of influenza and COVID-19 vaccines was administered to a representative sample of the Jewish Israeli population (see S1 Appendix). The questionnaire was pretested by 5 people and was not validated.

### Ethical considerations

The study was approved by Wolfson Medical Center Internal Review board (0112-20-WOMC). As approved by the ethical committee, consent to participate in the study was given through the internet by agreeing to answer the questioner for research purposes. We maintained anonymity for all responders.

### Study design and sampling

Recruitment was designed to be a representative sample of the Jewish Israeli population excluding children under the age of 15 years, according to the definitions of the Israeli Central Bureau of Statistics with planned enrichment of the elderly population aged 65 years and older [23, 24]. We enriched the sample of people aged ≥ 65 years because they are prone to complications and death both from COVID-19 and influenza and we wanted to increase precision in this age group. The population thus was comprised of two samples a sample of people aged 16 to 64 years and a sample of people aged≥ 65 years. The size of each sample was calculated as follows:

$$\sqrt{Z_{0.95}*Z_{0.95}*(\frac{P_{0.5}*(1-P_{0.5})}{n})}$$

Where Z_0.95_ was set at 1.96. P_0.5_ was the maximal probability for the category. N was the sample size. This yielded a sample of 1,300 people aged < 65 years with a confidence interval (CI) of 2.72% and a sample size of 700 people aged ≥65 years with a CI of 2.72% both representing the Israeli population in the respective age groups. Quotas of responders for each cell were maintained according to age, sex, area of residence, and level of religiousness. Within each quota sampling was random.

### Data collection

Questionnaires were administered and all responses collected during the first week of September 2020. During this period, there was a significant rise in COVID-19 cases in Israel, but this was before the second Israeli lockdown that started September 25^th^, 2020, three weeks after the administration of the questionnaire.

The study was administered by the internet using the services of a survey company (Techmarketing) that employs an internet panel. Participants of this panel receive notifications regarding surveys by an application and by email inviting them to participate in a particular survey. The company administering the survey has basic information on participants from the Israeli Bureau of Statistics that enables the composition of a representative sample by cells according to age, sex, area of residence, and level of religiousness, thus ensuring the representativeness of the sample. For example, a particular cell can be defined as males, aged 18 to 20 years, from the center of Israel, who are secular. Once a particular cell is complete according to predefined quotas, no more people that fit the definition of this cell can respond to the survey. If certain cells were not filled, people who did not respond to the request to participate (agree or deny), were approached by phone. The subject of the survey was not known prior to accepting the invitation to respond to the survey. People who responded to the questionnaire received a small token of approximately $1.5. As the cohort maintained the structure of cells regarding age, sex, area of residence, and level of religiousness bias occurring due to people’s unwillingness to respond was assumed to be small. We do not have additional information regarding the non-responders. Anonymity of responders and non-responders was maintained.

Items in the questionnaire included age, sex, marital status, number of children, working status, level of income, level of religiousness, history of past vaccination to influenza, attitudes toward vaccination in the past and attitudes towards influenza vaccine in the coming winter season of 2020–2021. We also collected information regarding whether responders were infected with COVID-19, and if they had to be quarantined because of exposure to a known COVID-19 case. We also inquired if the COVID-19 situation affected their financial status. All responders were asked if they would get a vaccine against COVID-19 if available and if they were willing to participate in a clinical trial of a COVID-19 vaccine.

### Data analysis

We used Python and SPSS version 25 for statistical analysis. Categorical variables are presented as absolute number and percent, and continuous variables are presented as mean with standard deviations (SD) or median and intra-quartile range (IQR), as appropriate. The data were analyzed as a whole and according to the predefined groups (the entire cohort and responders aged <65 years and 65 years and older). Groups were compared using T test, Mann Whitney U test, Anova and Chi-Square test as appropriate. Willingness to vaccinate against influenza last year and this fall, and willingness to vaccinate against influenza and COVID-19 in the coming season (2020–2021) was compared using paired T test. Percent increase in willingness to receive influenza vaccine this winter (2020–2021) was compared to receiving influenza vaccine last winter (2019–2020) and was calculated as follows: (the number of people wanting to receive influenza vaccine/the number of people that received the vaccine last year x 100)-100.

Risk factors for not wanting to receive vaccines (influenza, and COVID-19 vaccine) in the coming winter (2020–2021) were assessed using logistic regression for each vaccine. Independent variables found to be significantly associated with the dependent variable in a bivariate analysis (p<0.1) were entered into multivariate logistic regression analysis, (backwards conditional), with results presented as odds ratio (OR) with a confidence interval (CI) of 95%. Statistical significance was set at p<0.05.

## Results

We approached 10,625 people, of these 2,080 were willing to respond (19%), and 2,024 completed the questionnaire (97.3%). One thousand three hundred and thirteen (64.9%) people were 15 to 64 years old and 711 (35.1%) were 65 years and older. The sample of people aged 65 years and older comprised 35.2% of responders and their relative contribution to the Israeli population is 11%. Both groups are representative of the Jewish Israeli population according to age, sex, area of residence, and level of religiousness. Characteristics of the responders are presented in Table 1. Of note, 37% had co-morbidities, and in the sample of people aged 65 years and more 64% had comorbidities. The most common co-morbidities were diabetes, hypertension and obesity. Only 32 (1.6%) responders were diagnosed with COVID-19, 27 (2.1%) in the sample of people aged 16–64 years and 5 (0.7%) in the sample of people aged ≥65 years.

**Table 1:** Characteristics of people responding to the questionnaire.

	All responders N = 2,024	Responders < 65 years N = 1,313 (64.9%)	Responders ≥ 65 years N = 711 (35.1%)
**Age–years, mean (SD)**	49.12 (19.49)	37.5 (13.82)	70.54 (4.57)
** • 15–17 years**	99 (4.9)	99 (7.5)	-
** • 18–20 years**	56 (2.8)	56 (4.3)	-
** • 21–30 years**	311 (15.4)	311 (23.7)	-
** • 31–40 years**	322 (15.9)	322 (24.5)	-
** • 41–50 years**	245 (12.1)	245 (18.7)	-
** • 51–64 years**	280 (13.8)	280 (21.3)	-
** • 65–74 years**	592 (29.2)	-	592 (83.3)
** • >74 years**	119 (5.9)	-	119 (16.7)
**Female sex n (%)**	1,053 (52.0)	675 (51.4)	378 (53.2)
**Married—n (%)**	1,169 (57.8)	694 (52.9)	475 (66.8)
**Orthodox Jew–n (%)**	145 (7.2)	138 (10.5)	7 (1.0)
**Secular—n (%)**	1,104 (54.5)	575 (43.8)	529 (74.4)
**Residence in central Israel–n (%)**	1,017 (50.2)	616 (46.9)	401 (56.4)
**University or college education–n (%)**	887 (43.8)	540 (41.1)	347 (48.8)
**Currently employed–n (%)**	1,048 (51.8%)	864 (65.8)	184 (25.9)
**Co-morbidities**	750 (37.0)	294 (22.4)	456 (64.0)
** • Diabetes—n (%)**	234 (11.6)	69 (5.3)	165 (23.2)
** • Hypertension—n (%)**	420 (20.8)	121 (9.2)	299 (42.1)
** • Obesity–n (%)**	304 (15.0%)	152 (11.6)	152 (21.4)
** • Heart disease–n (%)**	121 (6.0)	20 (1.5)	101 (14.2)
** • Lung disease–n (%)**	68 (3.4)	41 (3.1)	27 (3.8)
** • Malignancy = n (%)**	46 (2.3)	9 (0.7)	37 (5.2)
**Diagnosed with COVID-19 –n (%)**	32 (1.6)	27 (2.1)	5 (0.7)
**Were isolated once or more due to exposure to a confirmed COVID-19 case -n (%)**	387 (19.1)	329 (25.1)	58 (8.2)
**Financial status was compromised due to the COVID-19 pandemic–n (%)**	881 (43.5)	648 (49.4)	233 (32.8)

**Fig 1:**
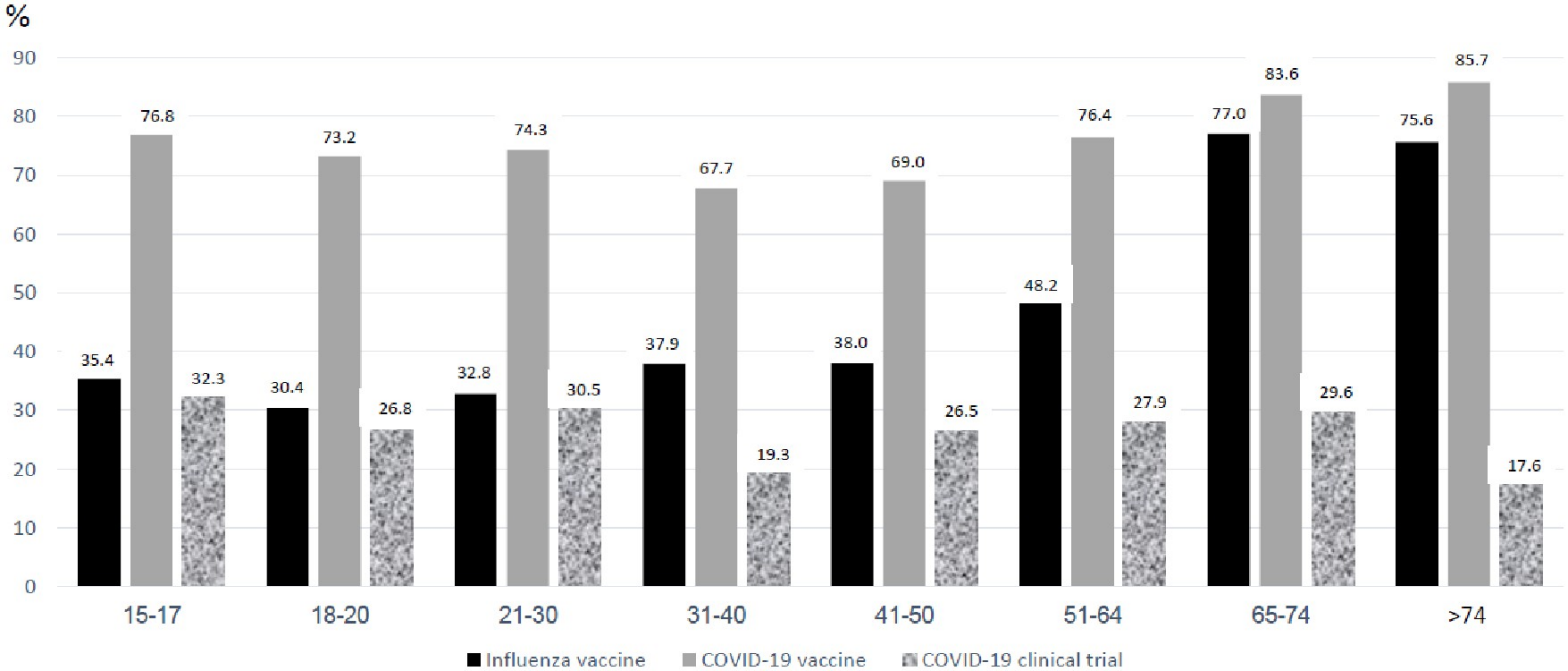
Relation between age and willingness to receive influenza vaccine this coming fall (2020–2021) and COVID-19 vaccine when available.

Attitudes towards Influenza, and COVID-19 vaccines are presented in Table 2. Main reasons for vaccine hesitancy in both samples were judging influenza vaccine to be ineffective (27.1%), and fear of major side effects (29.3%). We observed an increase of 14.1% in the willingness to receive an influenza vaccine this coming winter (season 2020–2021) compared to self-reported vaccination rates last winter (season 2019–2020) in the entire cohort (p<0.0001 CI 0.047–0.081), an increase of 22.6% in people aged less than 65 years and an increase of 7.3% in people aged 65 years and older (p<0.0001, CI 0.048–0.093) and p<0.0001, CI 0.027–0.077, respectively). People in the ≥65 years cohort were significantly more inclined to receive the influenza vaccine compared to people aged less than 65 years (p<0.0001). The relation between age categories and willingness to receive the influenza vaccine is presented in Fig 1.

**Table 2:** Attitudes towards influenza, and COVID-19 vaccines.

	All responders N = 2,024	Responders < 65 years N = 1,313 (64.9%)	Responders ≥ 65 years N = 711 (35.1%)
**Received influenza vaccine at least once in the past–n (%)**	1334 (65.9)	752 (57.3)	582 (81.8)
**Received influenza vaccine last winter (2019–2020)–n (%)**	920 (45.5)	411 (31.3)	509 (71.6)
**Did not received influenza vaccine last winter (2019–2020)–n (%)**	1104 (54.5)	902 (68.7)	202 (28.4)
**Reasons for not getting influenza vaccine in the past winter (2019–2020)(responders could select all that apply)**			
** • Thinks influenza vaccine is ineffective–n (%)**	299 (27.1)	240 (26.6)	59 (29.2)
** • Thinks influenza vaccine causes major side effects–n (%)**	323 (29.3)	256 (28.4)	67 (33.2)
** • Fears injections–n (%)**	126 (11.4)	107 (11.9)	19 (9.4)
** • Unavailability of influenza vaccine–n (%)**	136 (12.3)	114 (12.6)	22 (10.9)
**Had an influenza like illness last winter–n (%)**	512 (25.3)	374 (28.5)	138 (19.4%)
**Refused to receive at least one vaccine over the course of one’s lifetime–n (%)**	206 (10.2)	108 (8.2)	98 (13.8)
**Opinions regarding vaccination in the coming flu season (2020–2021)**			
** • Thinks that this winter (2020–2021) influenza vaccine is more important–n (%)**	1122 (55.4)	626 (47.7)	496 (69.8)
** • Plans to receive influenza vaccine this coming winter (2020–2021)–n (%)**	1050 (51.9)	504 (38.4)	546 (76.8)
**Opinions regarding vaccination in the coming flu season**			
**Plans to receive COVID-19 vaccine when available–n (%)**	1548 (76.4)	949 (72.3)	597 (84.0)
**Willing to participate in a COVID-19 vaccine clinical trial–n (%)**	543 (26.8)	347 (26.4)	196 (27.6)

Willingness to receive COVID-19 vaccine when available was significantly higher than the willingness to receive an influenza vaccine in the coming winter (season 2020–2021), 942 (72.3%) in the 16–64 years cohort and 84.0% in the cohort aged ≥ 65 years (p<0.001, CI 0.309–0.369 and p<0.001, CI 0.045–0.101, respectively). One thousand five hundred and forty-eight people (76.4% of the cohort) wanted to receive the COVID-19 vaccine when available. Of these, 973 (62.9%) wanted to receive both the influenza vaccine and the COVID-19 vaccine. Of the 1,050 (51.9%) who planned to take the influenza vaccine, 973 (92.7%) wanted to also receive the COVID-19 vaccine when available.

Willingness to participate in trials assessing COVID-19 vaccines was much lower than the willingness to receive the COVID-19 vaccine when available as can be seen in Fig 1. Of the entire cohort, 543 people (26.8%) were willing to participate in a COVID-19 vaccine trial, and this was stable across all age groups.

We found that receipt of influenza vaccine last winter (season 2019–2020), obesity, malignancy, and age ≥65 years were significantly associated with planning to vaccinate against influenza this season (2020–2021) while refusal to receive a vaccine in the past was negatively associated with plans to receive the influenza vaccine (Table 3). Factors associated with wanting to receive the COVID-19 vaccine when available were different and included planning to take the influenza vaccine and being secular. Factors negatively associated with wanting to vaccinate against COVID-19 were refusal in the past to receive vaccines, and female sex (Table 4).

**Table 3:** Factors independently associated with plan to receive influenza vaccine this coming winter (2020–2021).

	N (%)	Univariate analysis, P value	Odds ratio	P value	95% Confidence interval
**Age >65 years**	547 (76.7)	<0.0001	2.591	<0.001	1.958–3.429
**Female sex**	532 (50.5)	0.182			
**Family status (married)**	661 (56.5)	<0.0001			
**Working**	468 (44.7)	<0.0001			
**Financial status affected by COVID-19**	619 (54.1)	0.017			
**Secular**	654 (59.2)	<0.0001			
**Diabetes**	178 (76.1)	<0.0001			
**Hypertension**	311 (74.0)	<0.0001			
**Obesity**	202 (66.2)	<0.0001	1.441	0.046	1.007–2.062
**Heart disease**	92 (76.0)	<0.0001			
**Lung disease**	38 (55.9)	0.502			
**Malignancy**	39 (84.8)	<0.0001	2.806	0.048	1.011–7.783
**Was isolated due to COVID-19**	267 (43.2)	<0.0001			
**Was diagnosed with COVID-19**	21 (65.6)	0.121			
**Received influenza vaccine last winter (2019–2020)**	826 (89.7)	<0.0001	26.113	<0.0001	20.059–33.995
**Refused to receive at least one vaccine over the course of one’s lifetime**	81 (39.3)	<0.0001	0.595	0.010	0.401–0.882
**Had influenza like illness last year (2019–2020)**	246 (48.0)	0.041			
**Constant (B**_**0**_ **)**			0.208	<0.001	

*N (%) of responders who plan to receive influenza vaccine this coming winter (2020–2021).

Nagelkerke R square– 0.573. Constant (B_0_)—the intercept of the regression line with the y -axis. Candidate variables: work status, was isolated due to exposure to a COVID-19 case, was diagnosed with COVID-19, financial status was compromised due to the COVID-19 pandemic, received the influenza vaccine last winter (2019–2020), refused to receive at least one vaccine over the course of one’s lifetime, diabetes, obesity, hypertension, heart disease, lung disease, malignancy, female sex, family status, age ≥65, secular. To enter the multivariable analyses p was set at <0.1.

**Table 4:** Factors independently associated with plans to receive COVID-19 vaccine when available.

	*N (%)	Univariate analysis, P value	Odds ratio	P value	95% Confidence interval
**Age >65 years**	599 (84.0)	<0.0001			
**Female sex**	763 (72.4)	<0.0001	0.629	<0.001	0.500–0.791
**Family status**	920 (78.6)	0.140			
**Working**	771 (73.6)	<0.0001			
**Financial status affected by COVID-19**	893 (78.0)	0.054			
**Secular**	887 (80.3)	<0.0001	1.294	0.028	1.029–1.627
**Diabetes**	206 (88.0)	<0.0001			
**Hypertension**	364 (86.7)	<0.0001			
**Obesity**	247 (81.0)	0.042			
**Heart disease**	105 (86.8)	0.007			
**Lung disease**	49 (72.1)	0.391			
**Malignancy**	41 (89.1)	0.047			
**Was isolated due to COVID-19**	277 (71.6)	0.013			
**Was diagnosed with COVID-19**	23 (71.9)	0.544			
**Received influenza vaccine last winter (2019–2020)**	825 (89.6)	<0.0001			
**Refused to receive at least one vaccine over the course of one’s lifetime**	121 (58.7)	<0.0001	0.444	<0.001	0.318–0.621
**Plans to receive influenza vaccine this coming fall (2020–2021)**	974 (92.7)	<0.0001	8.401	<0.001	6.422–10.990
**Constant (B**_**0**_ **)**			1.786	<0.0001	

*N (%) of responders who plan to receive COVID-19 vaccine when available.

Nagelkerke R square– 0.261. Constant (B_0_)—the intercept of the regression line with the y-axis. Candidate variables: was isolated due to exposure to a COVID-19 case, was diagnosed with COVID-19, financial status was compromised due to the COVID-19 pandemic, plans to receive influenza vaccine this winter (2020–2021), refused to receive at least one vaccine in the past, diabetes, obesity, hypertension, heart disease, lung disease, malignancy, female sex, family status, work status, age ≥65, secular. To enter the multivariable analyses p was set at <0.1.

## Discussion

This study demonstrates that opinions regarding vaccines in a representative sample of the Jewish Israeli population differ between influenza vaccines and COVID-19 vaccine. Only 51.9% of responders declared that they plan to receive the influenza vaccine this season (2020–2021), an increase of 14.1% compared to last season (2019–2020), while 76.4% declared that they want to receive the COVID-19 vaccine when available. Last year’s vaccination rates (season 2019–2020) for influenza in this cohort were only 45.5% even though influenza vaccines are available free of charge to all Israeli citizens. These results demonstrate significant vaccine hesitancy. The questionnaire was administered during the first week of September 2020, when Israel was experiencing the second wave of COVID-19 pandemic and after one lockdown due to COVID-19. At the time the questionnaire was administered data regarding the efficacy of COVID-19 vaccines were not available, nor was there information regarding the safety profile of the vaccines against COVID-19. The acceptance of the COVID-19 vaccine in our cohort was higher than that reported in a representative sample of the U.S. population [25], but still one in four individuals expressed vaccine hesitancy towards the COVID-19 vaccines and one in two against the influenza vaccine. In a survey conducted in the UK among 1,500 adults 64% reported they intend to vaccinate against COVID-19 when the vaccine will be available. Like our results, past vaccination against influenza was a strong predictor of wanting to receive COVID-19 vaccines [26]. These results are surprising. The study was conducted during a phase of sharply increasing COVID-19 illness in Israel when the Israeli public was already aware of the devastating effect of COVID-19 illness, both on mortality and morbidity. The additional costs of COVID-19 pandemic were also known at this time, such as closing of the educational system for many months, and significant effects on work status and economics. Many restaurants and all theatres and music venues were closed from March 14^th^ 2020, to May 27^th^ 2020, and from July 3^rd^ 2020 to August 14^th^ 2020. Many restrictions upon travelling to other countries were in place since April 2020 and were still ongoing when the survey was administered. In our study 43.5% declared that they were financially affected by the COVID-19 situation. Only a small number of responders were diagnosed with COVID-19, but 19.1% experienced at least one quarantine due to exposure to a COVID-19 patient. Yet, it should be stressed that the decision on whether to accept vaccination is a personal judgement on benefit versus risk. While the generic risk of catching COVID-19 was available in lay terms, neither the protective rate nor the risk rate regarding COVID-19 vaccines were available to respondents at the time the questionnaire was administered. Thus, responders were replying in the abstract without validated knowledge.

The strongest correlate of wanting to receive the COVID-19 vaccine was the wish to receive influenza vaccine this winter (2020–2021) and receipt of influenza vaccines in the past (data not shown). Refusal of any vaccines in the past was a strong negative correlate of planning to receive both vaccines. Thus, it seems that vaccine hesitancy is a state of mind against vaccines in general. In a study that mapped vaccine confidence across 149 countries, between 2015 and 2019, confidence in the importance of vaccines (rather than in their safety or effectiveness) was the strongest association with vaccine uptake compared with other determinants considered [27]. Another study demonstrated that vaccine hesitancy to the COVID-19 vaccine is related to mistrust of vaccine benefit, and to worry about unforeseen future effects, concerns about commercial profiteering from pharmaceutical companies, and preferences for natural immunity [28].

Some correlates differed between hesitancy toward the COVID-19 vaccine and influenza vaccine. Comorbidities (obesity and malignancy) and age 65 years and older were independently associated with wanting to vaccinate against influenza whereas these comorbidities in the same cohort were not significantly associated with wanting to receive the COVID-19 vaccine. The wish to receive COVID-19 vaccine was stable across all age groups even though severity of disease and risk of death from COVID-19 are highly correlated to older age and presence of comorbidities [29]. Specific determinants of vaccine hesitancy for COVID-19 were religiousness and female sex. Previous studies have demonstrated a correlation between some religious groups and lower probabilities of vaccine uptake [29, 30]. Of note, religiousness varied by age and was more prevalent in younger responders. This is in accordance with data published by the Israeli Central bureau of Statistics and reflects changing trends in the Israeli Society [23, 24]. Regarding the association between sex and acceptance of vaccines, a previous publication demonstrated that male sex is associated with vaccine hesitancy in general [31]. A study from France also demonstrated that females were more hesitant to receive the COVID-19 vaccine compared to men [32]. In another study performed in a cohort of elderly home-bound citizens in New York, female sex was associated with hesitancy toward influenza vaccine [33]. Thus, sex plays a complicated role in vaccine hesitancy and further study is needed to understand this association. Despite previous reports on the association of affluence, both financial and educational, and vaccine hesitancy [34, 35], in the Israeli cohort neither financial status nor education level were independently associated with vaccine hesitancy. Understanding barriers to vaccine acceptance is important, and it is important to distinguish between generic barriers to all vaccines and barriers related to specific vaccine types and products to design effective programs enhancing vaccine acceptance.

We identified a significant difference between the wish to receive the COVID-19 vaccine (76.4% of the cohort) and wanting to participate in clinical trials assessing vaccine effectiveness and safety (26.8% of the cohort). It is reasonable to assume that the actual real-life acceptance of the COVID-19 vaccines will depend on how individuals perceive the vaccines on the spectrum between experimental vaccines to a vaccine with sound evidence regarding efficacy and safety. Public discussion regarding the level of evidence, and the way of disseminating the evidence to the public may have a significant effect on COVID-19 vaccine acceptance. Our results demonstrate that not all opinions regarding vaccines rely on sound evidence. Even though influenza vaccines have been available in Israel for many years, and the safety profile of influenza vaccines is good, hesitancy toward this vaccine was much higher than toward the COVID-19 vaccines. This may be related to the perception that influenza is a milder illness compared to COVID-19, and/or, to the perceptions that influenza vaccines are not effective and/or not safe. Reasons for influenza vaccine hesitancy were varied.

This study has several limitations. The study population was a representative sample of the Israeli Jewish population. The response rate was only 19% so this may have biased the population responding to the survey. The opinions of this cohort may differ from opinions in other communities and countries. The results represent the opinions of a representative sample of the Jewish Israeli population during the first week of September 2020 and these opinions may shift as the pandemic develops locally and globally, and as additional data regarding the coming influenza season (2020–2021) are available, and as more data are available regarding the COVID-19 vaccines. Due to concerns that we could not ensure the representativeness of non-Jewish participants in this sampling method and account for selection bias they were not included in the survey. Little information is present on barriers to vaccination in the Arab population in Israel. Significant Influenza vaccine hesitancy was observed in Arabs from Eastern Jerusalem [36], thus this important topic requires further study.

In conclusion, in this representative cohort of the Jewish Israeli population, vaccine hesitancy was common, one in two people were hesitant to receive influenza vaccine and one in four were hesitant to receive the COVID-19 vaccine. We identified generic as well as specific factors affecting vaccine hesitancy that may help to develop strategies to overcome vaccine hesitancy. The results point to areas of fear and lack of knowledge that can be addressed by better education of the public and by increasing trust between physicians and the community to increase vaccine acceptance.

## Supporting information

S1 Appendix (DOC)

## References

[1] Iuliano AD: Roguski KM, Chang HH, et al. Global Seasonal Influenza-associated Mortality Collaborator Network Estimates of global seasonal influenza-associated respiratory mortality: a modelling study. Lancet. 2018;391(10127):1285. Epub 2017 Dec 14. doi: 10.1016/S0140-6736(17)33293-2 29248255 PMC5935243

[2] Thompson WW, Shay DK, Weintraub E, Brammer L, Bridges CB, Cox NJ, et al. Influenza-associated hospitalizations in the United States. JAMA. 2004;292(11):1333. doi: 10.1001/jama.292.11.1333 15367555

[3] Neuzil KM, Reed GW, Mitchel EF Jr, Griffin MR. Influenza-associated morbidity and mortality in young and middle-aged women. JAMA. 1999;281(10):901. doi: 10.1001/jama.281.10.901 10078486

[4] Kilbourne ED. Influenza immunity: new insights from old studies. J Infect Dis. 2006;193(1):7. doi: 10.1086/498984 16323125

[5] Grohskopf LA, Alyanak E, Broder KR, Walter EB, Fry AM, Jernigan DB. Prevention and Control of Seasonal Influenza with Vaccines: Recommendations of the Advisory Committee on Immunization Practices—United States, 2019–20 Influenza Season. MMWR Recomm Rep. 2019;68(3):1. Epub 2019 Aug 23. doi: 10.15585/mmwr.rr6803a1 31441906 PMC6713402

[6] Henderson L, Millett C, Thorogood N. Perceptions of childhood immunization in a minority community: qualitative study. J R Soc Med. 2008 May;101(5):244–51 doi: 10.1258/jrsm.2008.070363 18463280 PMC2376260

[7] World Health Organization. Coronavirus disease (COVID-19) Weekly Epidemiological Update Data as received by WHO from national authorities, as of 10 am CEST 30 August 2020.

[8] Cao B, Wang Y, Wen D, et al. A trial of lopinavir-ritonavir in adults hospitalized with severe Covid-19 N Engl J Med. 2020 May 7;382(19):1787–1799. doi: 10.1056/NEJMoa2001282 32187464 PMC7121492

[9] Geleris J, Sun Y, Platt J, et al. Observational study of Hydroxychloroquine in Hospitalized Patients with COVID-19. N Engl J Med. 2020 May 7. [Epub ahead of print]. doi: 10.1056/NEJMoa2012410 32379955 PMC7224609

[10] Grein J, Ohmagari N, Shin D et al. Compassionate use of remdesivir for patients with severe Covid-19. New Engl J Med 2020, April 10. [Epub ahead of print]. doi: 10.1056/NEJMoa2007016 32275812 PMC7169476

[11] Wang Y, Zhang D, Du G et al. Remdesivir in adults with severe COVID-19: a randomised, double-blind, placebo-controlled, multicentre trial. Lancet 2020, April. [Epub ahead of print]. doi: 10.1016/S0140-6736(20)31022-9 32423584 PMC7190303

[12] van der Plas JL, Roestenberg M, Cohen AF, Kamerling IMC. How to expedite early phase SARS-CoV-2 vaccine trials in pandemic setting—a practical perspective. Br J Clin Pharmacol. 2020 Jun 19. Online ahead of print. doi: 10.1111/bcp.14435 32557771 PMC7300705

[13] Sondagar C, Xu R, MacDonald NE, Dubé E. Vaccine acceptance: How to build and maintain trust in immunization. Can Commun Dis Rep. 2020 May 7;46(5):155–159. doi: 10.14745/ccdr.v46i05a09 32558811 PMC7279131

[14] Glatman-Freedman A, Amir K, et al. Factors associated with childhood influenza vaccination in Israel: a cross-sectional evaluation. Isr J Health Policy Res. 2019 Nov 26;8(1):82. doi: 10.1186/s13584-019-0349-x 31771629 PMC6878635

[15] Stein-Zamir C, Israeli A. Timeliness and completeness of routine childhood vaccinations in young children residing in a district with recurrent vaccine-preventable disease outbreaks, Jerusalem, Israel. Euro Surveill. 2019 Feb;24(6):1800004 10.2807/1560-7917.ES.2019.24.6.1800004 PMC6373067 30755293

[16] Roller-Wirnsberger R, Lindner S, Kolosovski L, Platzer E, Dovjak P, Flick H, et al. The role of health determinants in the influenza vaccination uptake among older adults (65+): a scope review. Aging Clin Exp Res. 2021 Feb 15:1–10. Epub ahead of print. doi: 10.1007/s40520-021-01793-3 33587270 PMC7882864

[17] González-Block MÁ, Gutiérrez-Calderón E, Pelcastre-Villafuerte BE, Arroyo-Laguna J, Comes Y, Crocco P, et al. Influenza vaccination hesitancy in five countries of South America. Confidence, complacency and convenience as determinants of immunization rates. PLoS One. 2020 Dec 11;15(12):e0243833. doi: 10.1371/journal.pone.0243833 33306744 PMC7732123

[18] Verger P, Dualé C, Lenzi N, Scronias D, Pulcini C, Launay O. Vaccine hesitancy among hospital staff physicians: A cross-sectional survey in France in 2019. Vaccine. 2021 Jun 28:S0264-410X(21)00803-3. Epub ahead of print. doi: 10.1016/j.vaccine.2021.06.053 34210575

[19] Wake AD. The Willingness to Receive COVID-19 Vaccine and Its Associated Factors: "Vaccination Refusal Could Prolong the War of This Pandemic"—A Systematic Review. Risk Manag Healthc Policy. 2021 Jun 21;14:2609–2623. doi: 10.2147/RMHP.S311074 34188572 PMC8232962

[20] Lissitsa S, Madar G. Do disabilities impede the use of information and communication technologies? Findings of a repeated cross-sectional study—2003–2015. Isr J Health Policy Res. 2018 Oct 26;7(1):66. doi: 10.1186/s13584-018-0260-x 30367657 PMC6204019

[21] Ganaim A. The Internet in the Arab society. The Israeli internet association (ISOC-L). January 2018. https://fs.knesset.gov.il/20/Committees/20_cs_bg_525635.pdf

[22] Population of Israel on the Eve of 2020. Central bureau of statistics. https://www.cbs.gov.il/he/mediarelease/Pages/2020/%D7%90%D7%95%D7%9B%D7%9C%D7%95%D7%A1%D7%99%D7%99%D7%AA-%D7%99%D7%A9%D7%A8%D7%90%D7%9C-%D7%91%D7%A4%D7%AA%D7%97%D7%94-%D7%A9%D7%9C-%D7%A9%D7%A0%D7%AA-2021-.aspx

[23] Central bureau of statistics. https://www.cbs.gov.il/en/Pages/default.aspx.

[24] Society in Israel. Religion and self-definition of level of religiosity. Report No. 10. June 2018.

[25] Fisher KA, Bloomstone BA, Walder J, Crawford S, Fouayzi H, Mazor KM. Attitudes Toward a Potential SARS-CoV2020 Jun 19. -2 Vaccine: A Survey of U.S. Adults. Ann Intern Med. 2020 Jun 19. Online ahead of print. 10.7326/M20-3569 PMC7505019 32886525

[26] Sherman SM, Smith LE, Sim J, Amlôt R, Cutts M, Dasch H, et al. COVID-19 vaccination intention in the UK: results from the COVID-19 vaccination acceptability study (CoVAccS), a nationally representative cross-sectional survey. Hum Vaccin Immunother. 2020 Nov 26;1–10. Online ahead of print. 33242386 10.1080/21645515.2020.1846397 PMC8115754

[27] de Figueiredo A, Simas C, Karafillakis E, Paterson P, Larson HJ. Mapping global trends in vaccine confidence and investigating barriers to vaccine uptake: a large-scale retrospective temporal modelling study. Lancet Sep 10, 2020. Online ahead of print. doi: 10.1016/S0140-6736(20)31558-0 32919524 PMC7607345

[28] Taylor S, Landry CA, Paluszek MM, Groenewoud R, Rachor GS, Asmundson GJG. A Proactive Approach for Managing COVID-19: The Importance of Understanding the Motivational Roots of Vaccination Hesitancy for SARS-CoV2. Front Psychol. 2020 Oct 19;11:575950. doi: 10.3389/fpsyg.2020.575950 33192883 PMC7604422

[29] Berenguer J, Ryan P, Rodríguez-Baño J, Jarrín I, Carratalà J, Pachón J, et al. Characteristics and predictors of death among 4035 consecutively hospitalized patients with COVID-19 in Spain. Clin Microbiol Infect 2020 Nov;26(11):1525–1536. doi: 10.1016/j.cmi.2020.07.024 32758659 PMC7399713

[30] Phadke VK, Bednarczyk RA, Salmon DA, Omer SB. Association between vaccine refusal and vaccine-preventable diseases in the United States: a review of measles and pertussis. JAMA 2016 Mar 15;315(11):1149–58. doi: 10.1001/jama.2016.1353 26978210 PMC5007135

[31] Rozbroj T, Lyons A, Lucke J. Psychosocial and demographic characteristics relating to vaccine attitudes in Australia. Patient Educ Couns 2019 Jan;102(1):172–179. doi: 10.1016/j.pec.2018.08.027 30166057

[32] Detoc M, Bruel S, Frappe P, Tardy B, Botelho-Nevers E, Gagneux-Brunon A. Intention to participate in a COVID-19 vaccine clinical trial and to get vaccinated against COVID-19 in France during the pandemic. Vaccine 2020 Oct 21;38(45):7002–7006. doi: 10.1016/j.vaccine.2020.09.041 32988688 PMC7498238

[33] Banach DB, Ornstein K, Factor SH, Soriano GTA. Seasonal influenza vaccination among homebound elderly receiving home-based primary care in New York City. J Community Health 2012 Feb;37(1):10–4. doi: 10.1007/s10900-011-9409-z 21533885 PMC12422410

[34] McNutt LA, Desemone C, DeNicola E, Chebib HE, Nadeau JA, Bednarczyk RA, et al. Affluence as a predictor of vaccine refusal and under immunization in California private kindergartens. Vaccine 2016 Mar 29;34(14):1733–8. doi: 10.1016/j.vaccine.2015.11.063 26679403

[35] Özceylan G, Toprak D, Esen ES. Vaccine rejection and hesitation in Turkey. Hum Vaccin Immunother 2020 May 3;16(5):1034–1039. doi: 10.1080/21645515.2020.1717182 32027218 PMC7227707

[36] Southern J, Roizin H, Daana M, et al. Varied utilisation of health provision by Arab and Jewish residents in Israel. Int J Equity Health 2015 Aug 7;14:63. doi: 10.1186/s12939-015-0193-8 26245327 PMC4527256

