# Supplementary material for: Attitudes towards influenza, and COVID-19 vaccines during the COVID-19 pandemic among a representative sample of the Jewish Israeli population
Source: PLoS One. 2022 Feb 11;17(2):e0255495. doi: 10.1371/journal.pone.0255495 (PMC8836312; doi:10.1371/journal.pone.0255495)
Supplement: S1 Appendix — (DOC) [file pone.0255495.s001.doc]

**סקר עמדות לחיסון כנגד שפעת וחיסון כנגד נגיף הקורונה החדש (COVID-19)***

הסקר הועבר בפלטפורמה אינטרנטית.

שלום, שמי ד"ר יסמין מאור ואני מנהלת את היחידה למחלות זיהומיות במרכז הרפואי וולפסון.

אני מעוניינת להבין את עמדות הציבור לגבי חיסונים כנגד מחלות שונות ולכן אשמח מאד עם תוכלו להקדיש זמן קצר למילוי הסקר הזה.

השאלון הוא אנונימי. הנתונים נשמרים באופן בלתי מזוהה ולא משמשים לדבר מלבד מטרת השאלון.

השאלות מנוסחות בלשון זכר אולם מיועדות לנשים וגברים כאחד.

תודה רבה על שיתוף הפעולה!

אני מקווה כי התשובות יעזרו לנו להיערך טוב יותר לחורף הקרוב.

1. מהו הסטטוס שלך נכון להיום?

- תלמיד
- חייל
- סטודנט
- שכיר
- עצמאי
- מובטל / בחל"ת
- עקר בית
- פנסיונר

1. האם יש לך ילדים מתחת לגיל 18? אם כן באילו טווחי גילאים? ניתן לסמן יותר מתשובה אחת. מולטי

- עד לגיל שנתיים
- 2-5
- 6-10
- 11-18
- לא, אין לי ילדים מתחת לגיל 18

| 1. אנא סמן האם וכמה פעמים עשית פעולות אלו: | | | | | |
| --- | --- | --- | --- | --- | --- |
|  |  | כן, פעם אחת | כן, פעמיים | כן, שלוש פעמים או יותר | לא |
|  | היית בבידוד עקב הקורונה |  |  |  |  |
|  | נבדקת לקורונה |  |  |  |  |

1. האם חלית בקורונה?

- כן
- לא

1. האם הושפעת כלכלית ממגפת הקורונה?

- מצבי הכלכלי הורע מאוד
- מצבי הכלכלי הורע במידה
- אין שינוי במצבי הכלכלי
- מצבי הכלכלי השתפר במידה
- מצבי הכלכלי השתפר מאוד

1. האם אתה מתכנן להתחסן בסתיו הקרוב לשפעת?

- כן
- לא
- עוד לא החלטתי

1. האם אי פעם התחסנת לשפעת?

- כן
- לא
- לא זוכר

1. האם בשנה שעברה התחסנת לשפעת?

- כן
- לא

1. האם יש חיסונים אחרים שהומלץ לך לקבל ולא התחסנת?

- כן
- לא

1. האם חלית בשנה שעברה בשפעת?

- כן
- אני חושב שכן אבל לא בוצעה לי בדיקה לשפעת
- לא

1. האם השנה יותר חשוב להתחסן לשפעת בהשוואה לשנה שעברה?

- כן
- לא
- לא יודע

1. האם מגפת הקורונה משפיעה על הרצון להתחסן לשפעת?

- בגלל מגפת הקורונה נראה לי יותר חשוב להתחסן השנה לשפעת
- למגפת הקורונה אין השפעה על ההחלטה שלי להתחסן לשפעת
- בגלל מגפת הקורונה החלטתי לא לקחת חיסון לשפעת השנה

תנאי כניסה לשאלה 13: השיב "לא" בשאלה 8

מה מהסיבות הבאות גרמו לך לא להתחסן לשפעת בשנה שעברה? ניתן לסמן יותר מתשובה אחת

- החיסון לא יעיל
- לחיסון תופעות לואי קשות
- אני פוחד מזריקות יותר משאני פוחד משפעת
- רציתי להתחסן אבל היה מחסור בחיסונים
- רציתי להתחסן אבל קופת החולים לא היתה פתוחה בשעות נוחות
- רציתי להתחסן אבל לא חיסנו במקום העבודה שלי
- אחר, אנא פרט: _____________

כולם

1. האם אתה סובל מאחת מהמחלות הבאות? (ניתן לסמן יותר מתשובה אחת)

- סוכרת
- יתר לחץ דם
- השמנת יתר
- מחלת לב
- מחלת ראות
- מחלה גידולית
- אינני סובל מאף אחת מהמחלות הלללו

1. האם אתה נוטל תרופות מדכאות חיסון?

- כן
- לא

1. במידה ויהיה חיסון לנגיף הקורונה החדש האם תבחר להתחסן?

- כן
- לא

1. במידה ויציעו לך להשתתף במחקר הבוחן חיסון לנגיף הקורונה החדש האם תרצה להשתתף בניסוי כזה?

- כן
- לא

* נתונים דמוגרפיים סופקו מנתוני הפאנל האינטרנטי: גיל, מין, מחוז, הכנסה משפחתית, השכלה, דתיות, סטטוס משפחתי.

**Attitudes against the Flu Vaccine and COVID-19 vaccines***

The survey was delivered through an internet platform.

Hello, my name is Dr. Yasmine Maor and I am the Head of the Infectious Diseases Unit at the Wolfson Medical Center.

I am interested in understanding the public's attitudes regarding vaccines against various diseases and therefore I would be very happy if you could devote a short time to filling out this survey.

The questionnaire is anonymous. The data is stored unidentified and is used solely for the purpose of the questionnaire.

The questions are formulated in the masculine form but are intended for both men and women.

Thank you so much for your cooperation!

I hope that the answers will help us prepare better for this winter.

1. What is your current main status?

- High school student
- Soldier
- College or University student
- Employee
- Self-employed worker
- Unemployed / on unpaid leave
- Housewife
- Pensioner

1. Do you have children under the age of 18? In what age ranges? You can mark more than one answer. Multi

- Up to 2 years old
- 2-5
- 6-10
- 11-18
- No, I don't have children under the age of 18.

| 1. Please check whether and how many times you have done these actions: | | | | | |
| --- | --- | --- | --- | --- | --- |
|  |  | Yes, once | Yes, twice | Yes, three or more times | No |
|  | - In isolation due to COVID-19 |  |  |  |  |
|  | - Tested for COVID-19 |  |  |  |  |

1. Were you diagnosed with COVID-19?

- Yes
- No

1. Have you been economically affected by the COVID-19 pandemic?

- My economic situation has worsened greatly
- My economic situation has worsened to some extent
- There was no change in my economic situations
- My economic situation has improved to some extent
- My economic situation has improved greatly

1. Are you planning to get vaccinated this fall against the flu?

- Yes
- No
- I haven't decided yet

1. Have you ever been vaccinated against the flu?

- Yes
- No
- I can't remember

1. Did you get the flu vaccine last year?

- Yes
- No

1. Are there any other vaccines you refused to receive in the past?

- Yes
- No

1. Were you sick with the flu last year?

- Yes
- I think so, but I didn't have a flu test
- No

1. Is it more important to get vaccinated against the flu this year than last year?

- Yes
- No
- I do not know

1. Does the COVID-19 pandemic affect your wish to get vaccinated against the flu?

- Because of the COVID-19 pandemic, it seems to me more important to get the flu vaccine this year
- The COVID-19 pandemic has no effect on my decision to get vaccinated for the flu
- Due to the COVID-19 pandemic, I decided not to take the flu vaccine this year

Terms of entry to question13: Answer "no" in question 8

What were the reasons for your decision not to receive the flu vaccine last year? You can mark more than one answer

- The vaccine is ineffective
- The vaccine has severe side effects
- I'm more afraid of injections than I'm afraid of the flu
- I wanted to get vaccinated, but there was a lack of vaccines
- I wanted to get vaccinated, but my HMO wasn't open at convenient times
- I wanted to get vaccinated, but we didn't get the opportunity to vaccinate in my workplace
- Other, please list: ________________

Everybody

1. Do you have from one of the following diseases? (You can mark more than one answer)

- Diabetes
- Hypertension
- Obesity
- Heart disease
- Lung disease
- Cancer
- I do not have any of these conditions

1. Are you taking immunosuppressive medications?

- Yes
- No

1. If there will be a vaccine for COVID-19, will you choose to be vaccinated?

- Yes
- No

1. If you are offered to participate in a study examining a new vaccine for COVID-19, would you like to participate in such an experiment?

- Yes
- No

* Demographic data were provided from the internet panels data: age, gender, district, family income, education, religion, family status.
